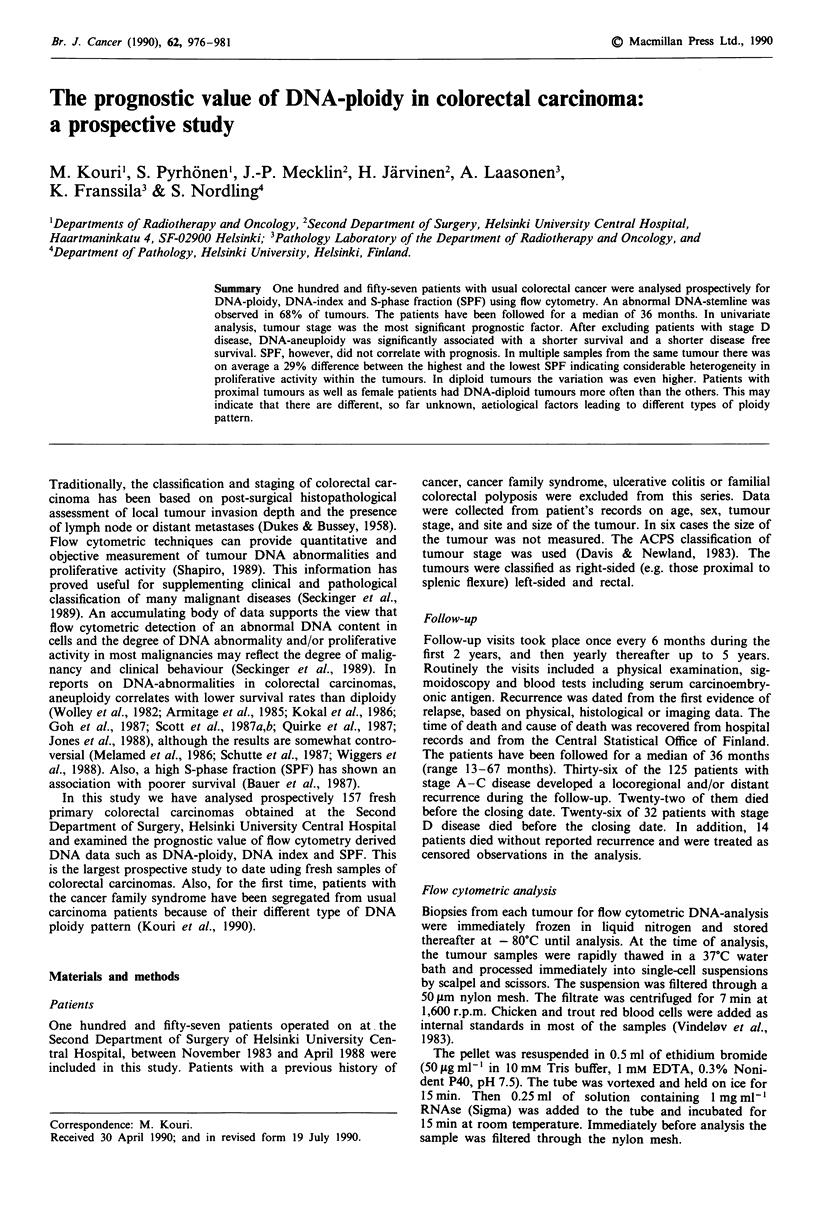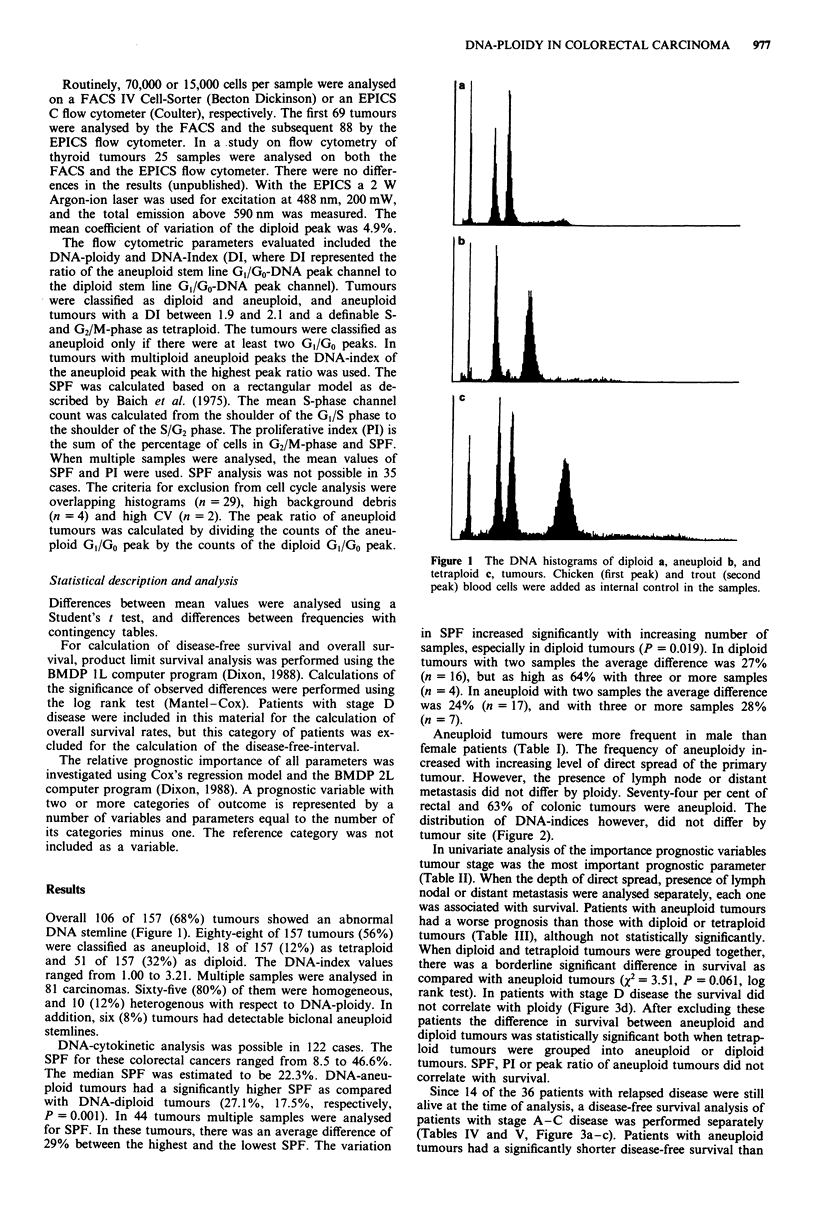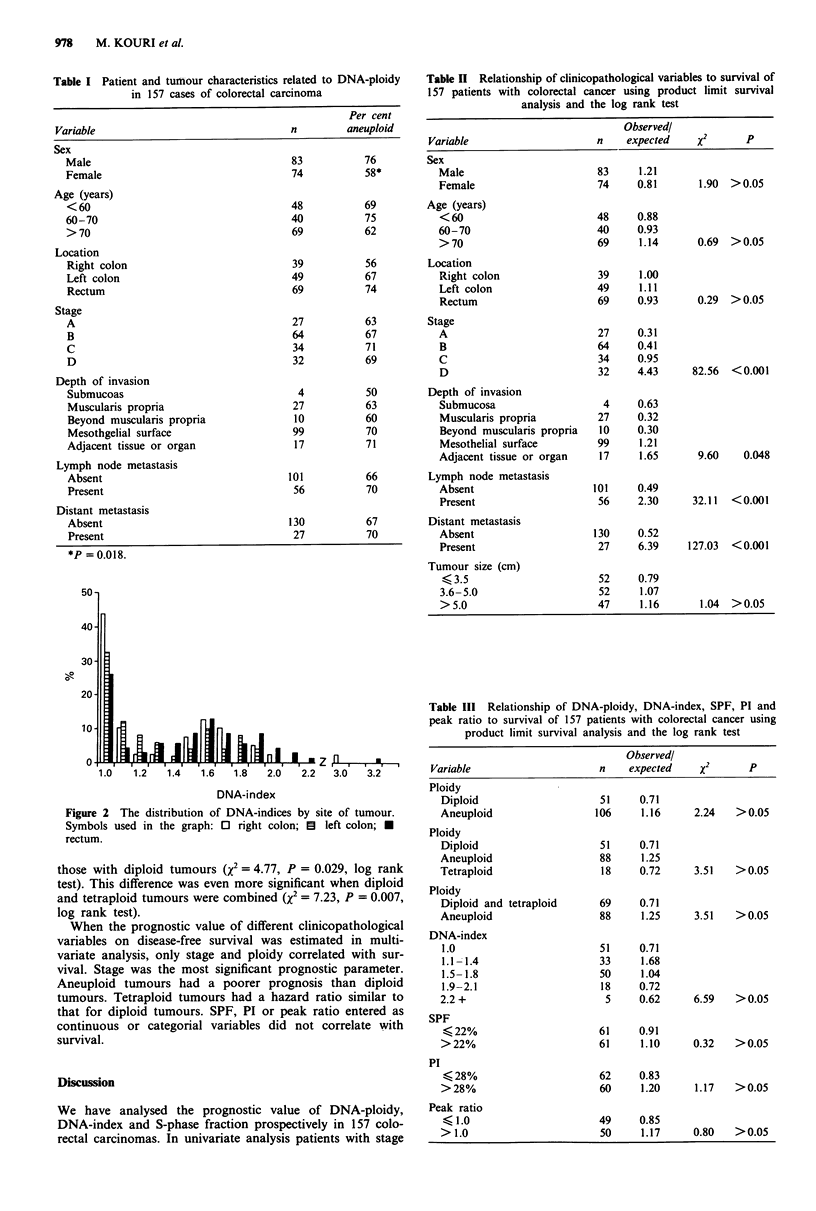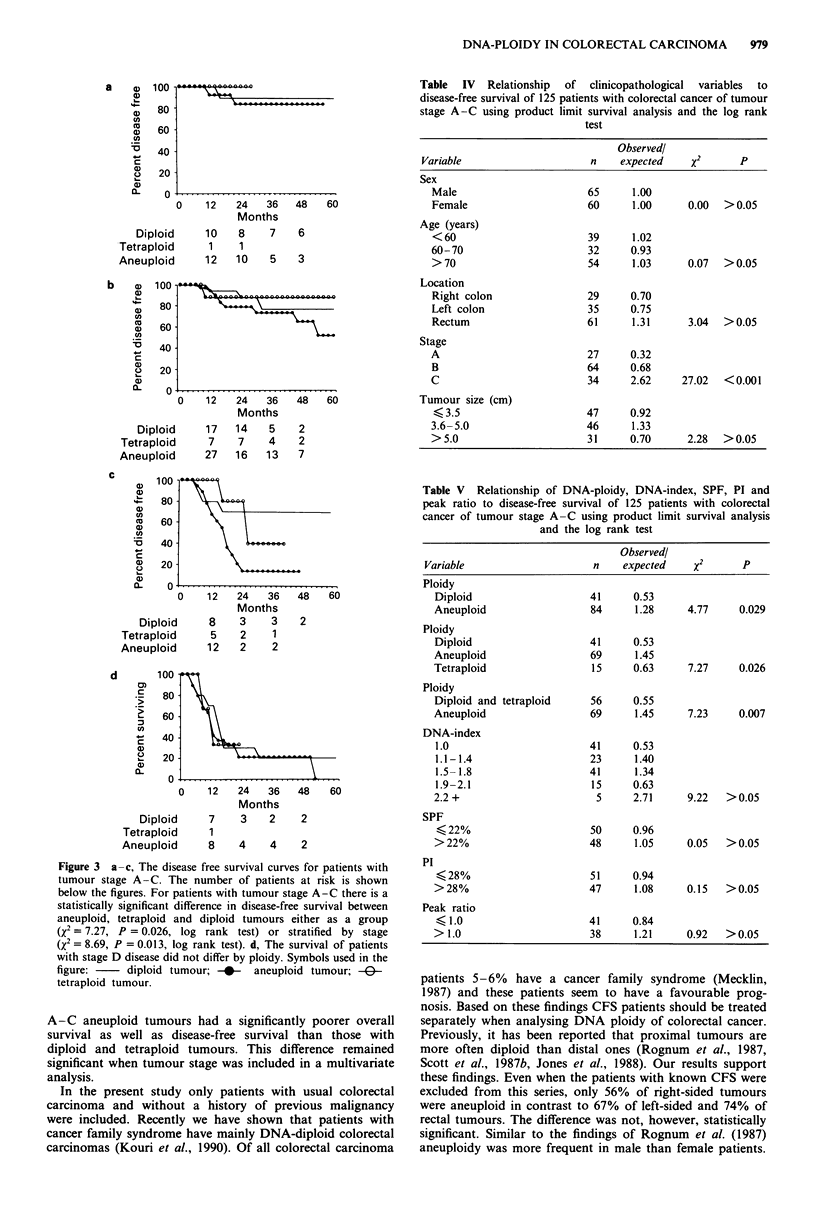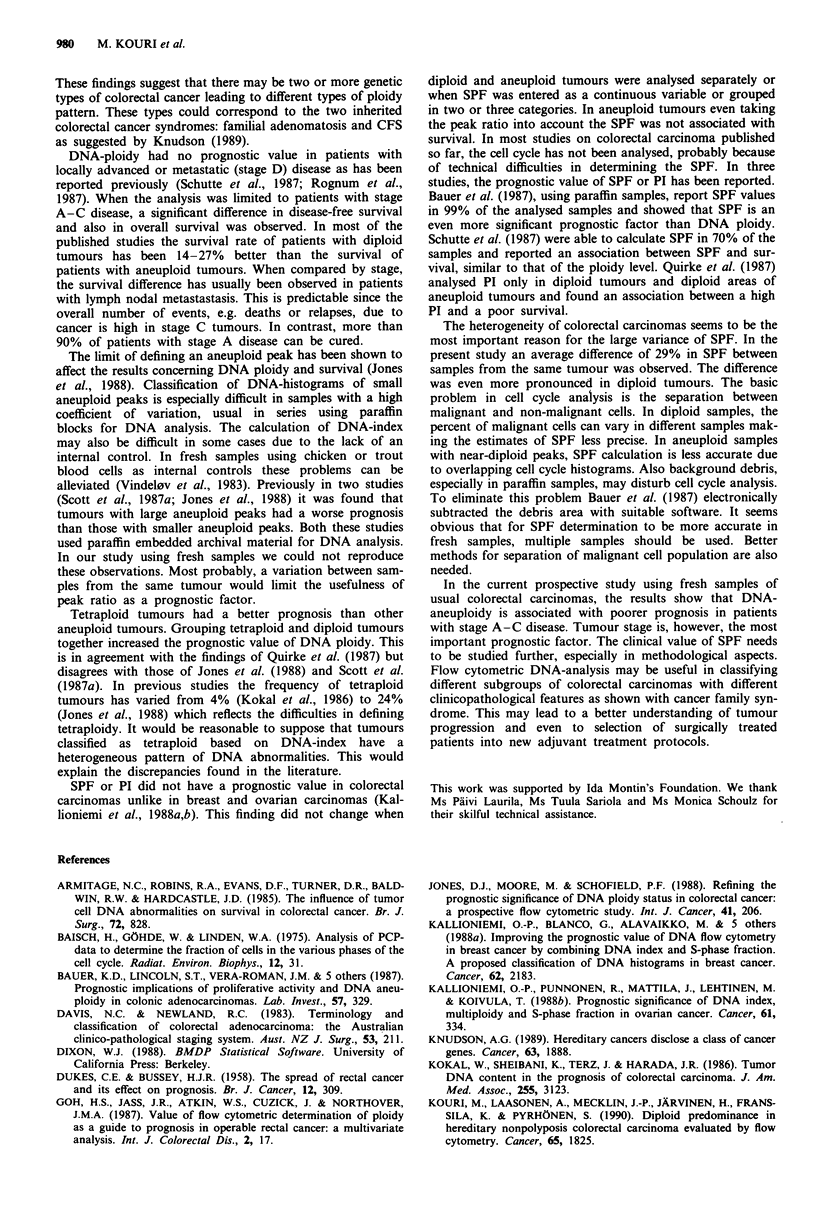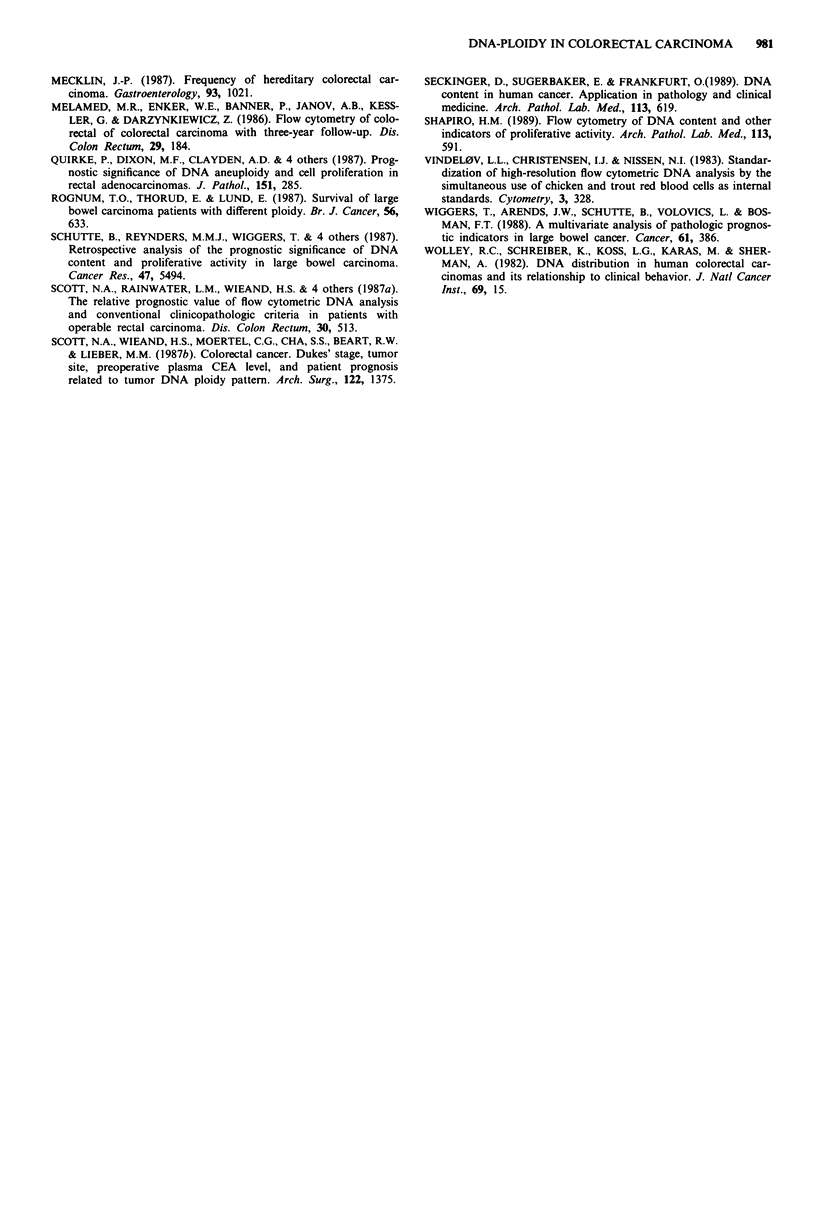# The prognostic value of DNA-ploidy in colorectal carcinoma: a prospective study.

**DOI:** 10.1038/bjc.1990.420

**Published:** 1990-12

**Authors:** M. Kouri, S. Pyrhönen, J. P. Mecklin, H. Järvinen, A. Laasonen, K. Franssila, S. Nordling

**Affiliations:** Department of Radiotherapy, Helsinki University Central Hospital, Finland.

## Abstract

One hundred and fifty-seven patients with usual colorectal cancer were analysed prospectively for DNA-ploidy, DNA-index and S-phase fraction (SPF) using flow cytometry. An abnormal DNA-stemline was observed in 68% of tumours. The patients have been followed for a median of 36 months. In univariate analysis, tumour stage was the most significant prognostic factor. After excluding patients with stage D disease, DNA-aneuploidy was significantly associated with a shorter survival and a shorter disease free survival. SPF, however, did not correlate with prognosis. In multiple samples from the same tumour there was on average a 29% difference between the highest and the lowest SPF indicating considerable heterogeneity in proliferative activity within the tumours. In diploid tumours the variation was even higher. Patients with proximal tumours as well as female patients had DNA-diploid tumours more often than the others. This may indicate that there are different, so far unknown, aetiological factors leading to different types of ploidy pattern.


Br. J. Cancer (1990), 62, 976-981 © Macmillan Press Ltd., 1990

### The prognostic value of DNA-ploidy in colorectal carcinoma: a prospective study

M. Kouri[1], S. Pyrhönen[1], J.-P. Mecklin[2], H. Järvinen[2], A. Laasonen[3], K. Franssila[3] & S. Nordling[4]

[1]*Departments of Radiotherapy and Oncology,* [2]*Second Department of Surgery, Helsinki University Central Hospital, Haartmaninkatu 4, SF-02900 Helsinki;* [3]*Pathology Laboratory of the Department of Radiotherapy and Oncology, and* [4]*Department of Pathology, Helsinki University, Helsinki, Finland.*

**Summary** One hundred and fifty-seven patients with usual colorectal cancer were analysed prospectively for DNA-ploidy, DNA-index and S-phase fraction (SPF) using flow cytometry. An abnormal DNA-stemline was observed in 68% of tumours. The patients have been followed for a median of 36 months. In univariate analysis, tumour stage was the most significant prognostic factor. After excluding patients with stage D disease, DNA-aneuploidy was significantly associated with a shorter survival and a shorter disease free survival. SPF, however, did not correlate with prognosis. In multiple samples from the same tumour there was on average a 29% difference between the highest and the lowest SPF indicating considerable heterogeneity in proliferative activity within the tumours. In diploid tumours the variation was even higher. Patients with proximal tumours as well as female patients had DNA-diploid tumours more often than the others. This may indicate that there are different, so far unknown, aetiological factors leading to different types of ploidy pattern.

Traditionally, the classification and staging of colorectal carcinoma has been based on post-surgical histopathological assessment of local tumour invasion depth and the presence of lymph node or distant metastases (Dukes & Bussey, 1958). Flow cytometric techniques can provide quantitative and objective measurement of tumour DNA abnormalities and proliferative activity (Shapiro, 1989). This information has proved useful for supplementing clinical and pathological classification of many malignant diseases (Seckinger *et al.*, 1989). An accumulating body of data supports the view that flow cytometric detection of an abnormal DNA content in cells and the degree of DNA abnormality and/or proliferative activity in most malignancies may reflect the degree of malignancy and clinical behaviour (Seckinger *et al.*, 1989). In reports on DNA-abnormalities in colorectal carcinomas, aneuploidy correlates with lower survival rates than diploidy (Wolley *et al.*, 1982; Armitage *et al.*, 1985; Kokal *et al.*, 1986; Goh *et al.*, 1987; Scott *et al.*, 1987*a,b*; Quirke *et al.*, 1987; Jones *et al.*, 1988), although the results are somewhat controversial (Melamed *et al.*, 1986; Schutte *et al.*, 1987; Wiggers *et al.*, 1988). Also, a high S-phase fraction (SPF) has shown an association with poorer survival (Bauer *et al.*, 1987).

In this study we have analysed prospectively 157 fresh primary colorectal carcinomas obtained at the Second Department of Surgery, Helsinki University Central Hospital and examined the prognostic value of flow cytometry derived DNA data such as DNA-ploidy, DNA index and SPF. This is the largest prospective study to date uding fresh samples of colorectal carcinomas. Also, for the first time, patients with the cancer family syndrome have been segregated from usual carcinoma patients because of their different type of DNA ploidy pattern (Kouri *et al.*, 1990).

### Materials and methods

### *Patients*

One hundred and fifty-seven patients operated on at the Second Department of Surgery of Helsinki University Central Hospital, between November 1983 and April 1988 were included in this study. Patients with a previous history of cancer, cancer family syndrome, ulcerative colitis or familial colorectal polyposis were excluded from this series. Data were collected from patient's records on age, sex, tumour stage, and site and size of the tumour. In six cases the size of the tumour was not measured. The ACPS classification of tumour stage was used (Davis & Newland, 1983). The tumours were classified as right-sided (e.g. those proximal to splenic flexure) left-sided and rectal.

### *Follow-up*

Follow-up visits took place once every 6 months during the first 2 years, and then yearly thereafter up to 5 years. Routinely the visits included a physical examination, sigmoidoscopy and blood tests including serum carcinoembryonic antigen. Recurrence was dated from the first evidence of relapse, based on physical, histological or imaging data. The time of death and cause of death was recovered from hospital records and from the Central Statistical Office of Finland. The patients have been followed for a median of 36 months (range 13-67 months). Thirty-six of the 125 patients with stage A-C disease developed a locoregional and/or distant recurrence during the follow-up. Twenty-two of them died before the closing date. Twenty-six of 32 patients with stage D disease died before the closing date. In addition, 14 patients died without reported recurrence and were treated as censored observations in the analysis.

### *Flow cytometric analysis*

Biopsies from each tumour for flow cytometric DNA-analysis were immediately frozen in liquid nitrogen and stored thereafter at − 80°C until analysis. At the time of analysis, the tumour samples were rapidly thawed in a 37°C water bath and processed immediately into single-cell suspensions by scalpel and scissors. The suspension was filtered through a 50 μm nylon mesh. The filtrate was centrifuged for 7 min at 1,600 r.p.m. Chicken and trout red blood cells were added as internal standards in most of the samples (Vindeløv *et al.*, 1983).

The pellet was resuspended in 0.5 ml of ethidium bromide (50 μg $ml^{-1}$ in 10 mM Tris buffer, 1 mM EDTA, 0.3% Nonident P40, pH 7.5). The tube was vortexed and held on ice for 15 min. Then 0.25 ml of solution containing 1 mg $ml^{-1}$ RNAse (Sigma) was added to the tube and incubated for 15 min at room temperature. Immediately before analysis the sample was filtered through the nylon mesh.

Correspondence: M. Kouri.
Received 30 April 1990; and in revised form 19 July 1990.

Routinely, 70,000 or 15,000 cells per sample were analysed on a FACS IV Cell-Sorter (Becton Dickinson) or an EPICS C flow cytometer (Coulter), respectively. The first 69 tumours were analysed by the FACS and the subsequent 88 by the EPICS flow cytometer. In a study on flow cytometry of thyroid tumours 25 samples were analysed on both the FACS and the EPICS flow cytometer. There were no differences in the results (unpublished). With the EPICS a 2 W Argon-ion laser was used for excitation at 488 nm, 200 mW, and the total emission above 590 nm was measured. The mean coefficient of variation of the diploid peak was 4.9%.

The flow cytometric parameters evaluated included the DNA-ploidy and DNA-Index (DI, where DI represented the ratio of the aneuploid stem line $G_1/G_0$-DNA peak channel to the diploid stem line $G_1/G_0$-DNA peak channel). Tumours were classified as diploid and aneuploid, and aneuploid tumours with a DI between 1.9 and 2.1 and a definable S- and $G_2$/M-phase as tetraploid. The tumours were classified as aneuploid only if there were at least two $G_1/G_0$ peaks. In tumours with multiploid aneuploid peaks the DNA-index of the aneuploid peak with the highest peak ratio was used. The SPF was calculated based on a rectangular model as described by Baich *et al.* (1975). The mean S-phase channel count was calculated from the shoulder of the $G_1$/S phase to the shoulder of the S/$G_2$ phase. The proliferative index (PI) is the sum of the percentage of cells in $G_2$/M-phase and SPF. When multiple samples were analysed, the mean values of SPF and PI were used. SPF analysis was not possible in 35 cases. The criteria for exclusion from cell cycle analysis were overlapping histograms ($n$ = 29), high background debris ($n$ = 4) and high CV ($n$ = 2). The peak ratio of aneuploid tumours was calculated by dividing the counts of the aneuploid $G_1/G_0$ peak by the counts of the diploid $G_1/G_0$ peak.

### *Statistical description and analysis*

Differences between mean values were analysed using a Student's *t* test, and differences between frequencies with contingency tables.

For calculation of disease-free survival and overall survival, product limit survival analysis was performed using the BMDP 1L computer program (Dixon, 1988). Calculations of the significance of observed differences were performed using the log rank test (Mantel-Cox). Patients with stage D disease were included in this material for the calculation of overall survival rates, but this category of patients was excluded for the calculation of the disease-free-interval.

The relative prognostic importance of all parameters was investigated using Cox's regression model and the BMDP 2L computer program (Dixon, 1988). A prognostic variable with two or more categories of outcome is represented by a number of variables and parameters equal to the number of its categories minus one. The reference category was not included as a variable.

### Results

Overall 106 of 157 (68%) tumours showed an abnormal DNA stemline (Figure 1). Eighty-eight of 157 tumours (56%) were classified as aneuploid, 18 of 157 (12%) as tetraploid and 51 of 157 (32%) as diploid. The DNA-index values ranged from 1.00 to 3.21. Multiple samples were analysed in 81 carcinomas. Sixty-five (80%) of them were homogeneous, and 10 (12%) heterogenous with respect to DNA-ploidy. In addition, six (8%) tumours had detectable biclonal aneuploid stemlines.

DNA-cytokinetic analysis was possible in 122 cases. The SPF for these colorectal cancers ranged from 8.5 to 46.6%. The median SPF was estimated to be 22.3%. DNA-aneuploid tumours had a significantly higher SPF as compared with DNA-diploid tumours (27.1%, 17.5%, respectively, $P$ = 0.001). In 44 tumours multiple samples were analysed for SPF. In these tumours, there was an average difference of 29% between the highest and the lowest SPF. The variation

**Figure 1** The DNA histograms of diploid **a**, aneuploid **b**, and tetraploid **c**, tumours. Chicken (first peak) and trout (second peak) blood cells were added as internal control in the samples.

in SPF increased significantly with increasing number of samples, especially in diploid tumours ($P$ = 0.019). In diploid tumours with two samples the average difference was 27% ($n$ = 16), but as high as 64% with three or more samples ($n$ = 4). In aneuploid with two samples the average difference was 24% ($n$ = 17), and with three or more samples 28% ($n$ = 7).

Aneuploid tumours were more frequent in male than female patients (Table I). The frequency of aneuploidy increased with increasing level of direct spread of the primary tumour. However, the presence of lymph node or distant metastasis did not differ by ploidy. Seventy-four per cent of rectal and 63% of colonic tumours were aneuploid. The distribution of DNA-indices however, did not differ by tumour site (Figure 2).

In univariate analysis of the importance prognostic variables tumour stage was the most important prognostic parameter (Table II). When the depth of direct spread, presence of lymph nodal or distant metastasis were analysed separately, each one was associated with survival. Patients with aneuploid tumours had a worse prognosis than those with diploid or tetraploid tumours (Table III), although not statistically significantly. When diploid and tetraploid tumours were grouped together, there was a borderline significant difference in survival as compared with aneuploid tumours ($\chi^2$ = 3.51, $P$ = 0.061, log rank test). In patients with stage D disease the survival did not correlate with ploidy (Figure 3d). After excluding these patients the difference in survival between aneuploid and diploid tumours was statistically significant both when tetraploid tumours were grouped into aneuploid or diploid tumours. SPF, PI or peak ratio of aneuploid tumours did not correlate with survival.

Since 14 of the 36 patients with relapsed disease were still alive at the time of analysis, a disease-free survival analysis of patients with stage A-C disease was performed separately (Tables IV and V, Figure 3a-c). Patients with aneuploid tumours had a significantly shorter disease-free survival than

**Table I** Patient and tumour characteristics related to DNA-ploidy in 157 cases of colorectal carcinoma

| *Variable* | *n* | *Per cent aneuploid* |
|---|---|---|
| Sex | | |
| Male | 83 | 76 |
| Female | 74 | 58* |
| Age (years) | | |
| <60 | 48 | 69 |
| 60–70 | 40 | 75 |
| >70 | 69 | 62 |
| Location | | |
| Right colon | 39 | 56 |
| Left colon | 49 | 67 |
| Rectum | 69 | 74 |
| Stage | | |
| A | 27 | 63 |
| B | 64 | 67 |
| C | 34 | 71 |
| D | 32 | 69 |
| Depth of invasion | | |
| Submucoas | 4 | 50 |
| Muscularis propria | 27 | 63 |
| Beyond muscularis propria | 10 | 60 |
| Mesothgelial surface | 99 | 70 |
| Adjacent tissue or organ | 17 | 71 |
| Lymph node metastasis | | |
| Absent | 101 | 66 |
| Present | 56 | 70 |
| Distant metastasis | | |
| Absent | 130 | 67 |
| Present | 27 | 70 |

*$P = 0.018$.

**Figure 2** The distribution of DNA-indices by site of tumour. Symbols used in the graph: □ right colon; ⊟ left colon; ■ rectum.

those with diploid tumours ($\chi^2 = 4.77$, $P = 0.029$, log rank test). This difference was even more significant when diploid and tetraploid tumours were combined ($\chi^2 = 7.23$, $P = 0.007$, log rank test).

When the prognostic value of different clinicopathological variables on disease-free survival was estimated in multivariate analysis, only stage and ploidy correlated with survival. Stage was the most significant prognostic parameter. Aneuploid tumours had a poorer prognosis than diploid tumours. Tetraploid tumours had a hazard ratio similar to that for diploid tumours. SPF, PI or peak ratio entered as continuous or categorial variables did not correlate with survival.

### Discussion

We have analysed the prognostic value of DNA-ploidy, DNA-index and S-phase fraction prospectively in 157 colorectal carcinomas. In univariate analysis patients with stage

**Table II** Relationship of clinicopathological variables to survival of 157 patients with colorectal cancer using product limit survival analysis and the log rank test

| *Variable* | *n* | *Observed/ expected* | $\chi^2$ | *P* |
|---|---|---|---|---|
| Sex | | | | |
| Male | 83 | 1.21 | | |
| Female | 74 | 0.81 | 1.90 | >0.05 |
| Age (years) | | | | |
| <60 | 48 | 0.88 | | |
| 60–70 | 40 | 0.93 | | |
| >70 | 69 | 1.14 | 0.69 | >0.05 |
| Location | | | | |
| Right colon | 39 | 1.00 | | |
| Left colon | 49 | 1.11 | | |
| Rectum | 69 | 0.93 | 0.29 | >0.05 |
| Stage | | | | |
| A | 27 | 0.31 | | |
| B | 64 | 0.41 | | |
| C | 34 | 0.95 | | |
| D | 32 | 4.43 | 82.56 | <0.001 |
| Depth of invasion | | | | |
| Submucosa | 4 | 0.63 | | |
| Muscularis propria | 27 | 0.32 | | |
| Beyond muscularis propria | 10 | 0.30 | | |
| Mesothelial surface | 99 | 1.21 | | |
| Adjacent tissue or organ | 17 | 1.65 | 9.60 | 0.048 |
| Lymph node metastasis | | | | |
| Absent | 101 | 0.49 | | |
| Present | 56 | 2.30 | 32.11 | <0.001 |
| Distant metastasis | | | | |
| Absent | 130 | 0.52 | | |
| Present | 27 | 6.39 | 127.03 | <0.001 |
| Tumour size (cm) | | | | |
| ⩽3.5 | 52 | 0.79 | | |
| 3.6–5.0 | 52 | 1.07 | | |
| >5.0 | 47 | 1.16 | 1.04 | >0.05 |

**Table III** Relationship of DNA-ploidy, DNA-index, SPF, PI and peak ratio to survival of 157 patients with colorectal cancer using product limit survival analysis and the log rank test

| *Variable* | *n* | *Observed/ expected* | $\chi^2$ | *P* |
|---|---|---|---|---|
| Ploidy | | | | |
| Diploid | 51 | 0.71 | | |
| Aneuploid | 106 | 1.16 | 2.24 | >0.05 |
| Ploidy | | | | |
| Diploid | 51 | 0.71 | | |
| Aneuploid | 88 | 1.25 | | |
| Tetraploid | 18 | 0.72 | 3.51 | >0.05 |
| Ploidy | | | | |
| Diploid and tetraploid | 69 | 0.71 | | |
| Aneuploid | 88 | 1.25 | 3.51 | >0.05 |
| DNA-index | | | | |
| 1.0 | 51 | 0.71 | | |
| 1.1–1.4 | 33 | 1.68 | | |
| 1.5–1.8 | 50 | 1.04 | | |
| 1.9–2.1 | 18 | 0.72 | | |
| 2.2 + | 5 | 0.62 | 6.59 | >0.05 |
| SPF | | | | |
| ⩽22% | 61 | 0.91 | | |
| >22% | 61 | 1.10 | 0.32 | >0.05 |
| PI | | | | |
| ⩽28% | 62 | 0.83 | | |
| >28% | 60 | 1.20 | 1.17 | >0.05 |
| Peak ratio | | | | |
| ⩽1.0 | 49 | 0.85 | | |
| >1.0 | 50 | 1.17 | 0.80 | >0.05 |

**Figure 3** **a**–**c**, The disease free survival curves for patients with tumour stage A–C. The number of patients at risk is shown below the figures. For patients with tumour stage A–C there is a statistically significant difference in disease-free survival between aneuploid, tetraploid and diploid tumours either as a group ($\chi^2 = 7.27$, $P = 0.026$, log rank test) or stratified by stage ($\chi^2 = 8.69$, $P = 0.013$, log rank test). **d**, The survival of patients with stage D disease did not differ by ploidy. Symbols used in the figure: —— diploid tumour; —●— aneuploid tumour; —○— tetraploid tumour.

A–C aneuploid tumours had a significantly poorer overall survival as well as disease-free survival than those with diploid and tetraploid tumours. This difference remained significant when tumour stage was included in a multivariate analysis.

In the present study only patients with usual colorectal carcinoma and without a history of previous malignancy were included. Recently we have shown that patients with cancer family syndrome have mainly DNA-diploid colorectal carcinomas (Kouri *et al.*, 1990). Of all colorectal carcinoma patients 5–6% have a cancer family syndrome (Mecklin, 1987) and these patients seem to have a favourable prognosis. Based on these findings CFS patients should be treated separately when analysing DNA ploidy of colorectal cancer. Previously, it has been reported that proximal tumours are more often diploid than distal ones (Rognum *et al.*, 1987, Scott *et al.*, 1987*b*, Jones *et al.*, 1988). Our results support these findings. Even when the patients with known CFS were excluded from this series, only 56% of right-sided tumours were aneuploid in contrast to 67% of left-sided and 74% of rectal tumours. The difference was not, however, statistically significant. Similar to the findings of Rognum *et al.* (1987) aneuploidy was more frequent in male than female patients.

**Table IV** Relationship of clinicopathological variables to disease-free survival of 125 patients with colorectal cancer of tumour stage A–C using product limit survival analysis and the log rank test

| *Variable* | *n* | *Observed/ expected* | $\chi^2$ | *P* |
|---|---|---|---|---|
| Sex | | | | |
| Male | 65 | 1.00 | | |
| Female | 60 | 1.00 | 0.00 | >0.05 |
| Age (years) | | | | |
| <60 | 39 | 1.02 | | |
| 60–70 | 32 | 0.93 | | |
| >70 | 54 | 1.03 | 0.07 | >0.05 |
| Location | | | | |
| Right colon | 29 | 0.70 | | |
| Left colon | 35 | 0.75 | | |
| Rectum | 61 | 1.31 | 3.04 | >0.05 |
| Stage | | | | |
| A | 27 | 0.32 | | |
| B | 64 | 0.68 | | |
| C | 34 | 2.62 | 27.02 | <0.001 |
| Tumour size (cm) | | | | |
| ⩽3.5 | 47 | 0.92 | | |
| 3.6–5.0 | 46 | 1.33 | | |
| >5.0 | 31 | 0.70 | 2.28 | >0.05 |

**Table V** Relationship of DNA-ploidy, DNA-index, SPF, PI and peak ratio to disease-free survival of 125 patients with colorectal cancer of tumour stage A–C using product limit survival analysis and the log rank test

| *Variable* | *n* | *Observed/ expected* | $\chi^2$ | *P* |
|---|---|---|---|---|
| Ploidy | | | | |
| Diploid | 41 | 0.53 | | |
| Aneuploid | 84 | 1.28 | 4.77 | 0.029 |
| Ploidy | | | | |
| Diploid | 41 | 0.53 | | |
| Aneuploid | 69 | 1.45 | | |
| Tetraploid | 15 | 0.63 | 7.27 | 0.026 |
| Ploidy | | | | |
| Diploid and tetraploid | 56 | 0.55 | | |
| Aneuploid | 69 | 1.45 | 7.23 | 0.007 |
| DNA-index | | | | |
| 1.0 | 41 | 0.53 | | |
| 1.1–1.4 | 23 | 1.40 | | |
| 1.5–1.8 | 41 | 1.34 | | |
| 1.9–2.1 | 15 | 0.63 | | |
| 2.2+ | 5 | 2.71 | 9.22 | >0.05 |
| SPF | | | | |
| ⩽22% | 50 | 0.96 | | |
| >22% | 48 | 1.05 | 0.05 | >0.05 |
| PI | | | | |
| ⩽28% | 51 | 0.94 | | |
| >28% | 47 | 1.08 | 0.15 | >0.05 |
| Peak ratio | | | | |
| ⩽1.0 | 41 | 0.84 | | |
| >1.0 | 38 | 1.21 | 0.92 | >0.05 |

These findings suggest that there may be two or more genetic types of colorectal cancer leading to different types of ploidy pattern. These types could correspond to the two inherited colorectal cancer syndromes: familial adenomatosis and CFS as suggested by Knudson (1989).

DNA-ploidy had no prognostic value in patients with locally advanced or metastatic (stage D) disease as has been reported previously (Schutte et al., 1987; Rognum et al., 1987). When the analysis was limited to patients with stage A–C disease, a significant difference in disease-free survival and also in overall survival was observed. In most of the published studies the survival rate of patients with diploid tumours has been 14–27% better than the survival of patients with aneuploid tumours. When compared by stage, the survival difference has usually been observed in patients with lymph nodal metastastasis. This is predictable since the overall number of events, e.g. deaths or relapses, due to cancer is high in stage C tumours. In contrast, more than 90% of patients with stage A disease can be cured.

The limit of defining an aneuploid peak has been shown to affect the results concerning DNA ploidy and survival (Jones et al., 1988). Classification of DNA-histograms of small aneuploid peaks is especially difficult in samples with a high coefficient of variation, usual in series using paraffin blocks for DNA analysis. The calculation of DNA-index may also be difficult in some cases due to the lack of an internal control. In fresh samples using chicken or trout blood cells as internal controls these problems can be alleviated (Vindeløv et al., 1983). Previously in two studies (Scott et al., 1987a; Jones et al., 1988) it was found that tumours with large aneuploid peaks had a worse prognosis than those with smaller aneuploid peaks. Both these studies used paraffin embedded archival material for DNA analysis. In our study using fresh samples we could not reproduce these observations. Most probably, a variation between samples from the same tumour would limit the usefulness of peak ratio as a prognostic factor.

Tetraploid tumours had a better prognosis than other aneuploid tumours. Grouping tetraploid and diploid tumours together increased the prognostic value of DNA ploidy. This is in agreement with the findings of Quirke et al. (1987) but disagrees with those of Jones et al. (1988) and Scott et al. (1987a). In previous studies the frequency of tetraploid tumours has varied from 4% (Kokal et al., 1986) to 24% (Jones et al., 1988) which reflects the difficulties in defining tetraploidy. It would be reasonable to suppose that tumours classified as tetraploid based on DNA-index have a heterogeneous pattern of DNA abnormalities. This would explain the discrepancies found in the literature.

SPF or PI did not have a prognostic value in colorectal carcinomas unlike in breast and ovarian carcinomas (Kallioniemi et al., 1988a,b). This finding did not change when diploid and aneuploid tumours were analysed separately or when SPF was entered as a continuous variable or grouped in two or three categories. In aneuploid tumours even taking the peak ratio into account the SPF was not associated with survival. In most studies on colorectal carcinoma published so far, the cell cycle has not been analysed, probably because of technical difficulties in determining the SPF. In three studies, the prognostic value of SPF or PI has been reported. Bauer et al. (1987), using paraffin samples, report SPF values in 99% of the analysed samples and showed that SPF is an even more significant prognostic factor than DNA ploidy. Schutte et al. (1987) were able to calculate SPF in 70% of the samples and reported an association between SPF and survival, similar to that of the ploidy level. Quirke et al. (1987) analysed PI only in diploid tumours and diploid areas of aneuploid tumours and found an association between a high PI and a poor survival.

The heterogeneity of colorectal carcinomas seems to be the most important reason for the large variance of SPF. In the present study an average difference of 29% in SPF between samples from the same tumour was observed. The difference was even more pronounced in diploid tumours. The basic problem in cell cycle analysis is the separation between malignant and non-malignant cells. In diploid samples, the percent of malignant cells can vary in different samples making the estimates of SPF less precise. In aneuploid samples with near-diploid peaks, SPF calculation is less accurate due to overlapping cell cycle histograms. Also background debris, especially in paraffin samples, may disturb cell cycle analysis. To eliminate this problem Bauer et al. (1987) electronically subtracted the debris area with suitable software. It seems obvious that for SPF determination to be more accurate in fresh samples, multiple samples should be used. Better methods for separation of malignant cell population are also needed.

In the current prospective study using fresh samples of usual colorectal carcinomas, the results show that DNA-aneuploidy is associated with poorer prognosis in patients with stage A–C disease. Tumour stage is, however, the most important prognostic factor. The clinical value of SPF needs to be studied further, especially in methodological aspects. Flow cytometric DNA-analysis may be useful in classifying different subgroups of colorectal carcinomas with different clinicopathological features as shown with cancer family syndrome. This may lead to a better understanding of tumour progression and even to selection of surgically treated patients into new adjuvant treatment protocols.

This work was supported by Ida Montin's Foundation. We thank Ms Päivi Laurila, Ms Tuula Sariola and Ms Monica Schoulz for their skilful technical assistance.

### References

ARMITAGE, N.C., ROBINS, R.A., EVANS, D.F., TURNER, D.R., BALDWIN, R.W. & HARDCASTLE, J.D. (1985). The influence of tumor cell DNA abnormalities on survival in colorectal cancer. *Br. J. Surg.*, **72**, 828.

BAISCH, H., GÖHDE, W. & LINDEN, W.A. (1975). Analysis of PCP-data to determine the fraction of cells in the various phases of the cell cycle. *Radiat. Environ. Biophys.*, **12**, 31.

BAUER, K.D., LINCOLN, S.T., VERA-ROMAN, J.M. & 5 others (1987). Prognostic implications of proliferative activity and DNA aneuploidy in colonic adenocarcinomas. *Lab. Invest.*, **57**, 329.

DAVIS, N.C. & NEWLAND, R.C. (1983). Terminology and classification of colorectal adenocarcinoma: the Australian clinico-pathological staging system. *Aust. NZ J. Surg.*, **53**, 211.

DIXON, W.J. (1988). *BMDP Statistical Software*. University of California Press: Berkeley.

DUKES, C.E. & BUSSEY, H.J.R. (1958). The spread of rectal cancer and its effect on prognosis. *Br. J. Cancer*, **12**, 309.

GOH, H.S., JASS, J.R., ATKIN, W.S., CUZICK, J. & NORTHOVER, J.M.A. (1987). Value of flow cytometric determination of ploidy as a guide to prognosis in operable rectal cancer: a multivariate analysis. *Int. J. Colorectal Dis.*, **2**, 17.

JONES, D.J., MOORE, M. & SCHOFIELD, P.F. (1988). Refining the prognostic significance of DNA ploidy status in colorectal cancer: a prospective flow cytometric study. *Int. J. Cancer*, **41**, 206.

KALLIONIEMI, O.-P., BLANCO, G., ALAVAIKKO, M. & 5 others (1988a). Improving the prognostic value of DNA flow cytometry in breast cancer by combining DNA index and S-phase fraction. A proposed classification of DNA histograms in breast cancer. *Cancer*, **62**, 2183.

KALLIONIEMI, O.-P., PUNNONEN, R., MATTILA, J., LEHTINEN, M. & KOIVULA, T. (1988b). Prognostic significance of DNA index, multiploidy and S-phase fraction in ovarian cancer. *Cancer*, **61**, 334.

KNUDSON, A.G. (1989). Hereditary cancers disclose a class of cancer genes. *Cancer*, **63**, 1888.

KOKAL, W., SHEIBANI, K., TERZ, J. & HARADA, J.R. (1986). Tumor DNA content in the prognosis of colorectal carcinoma. *J. Am. Med. Assoc.*, **255**, 3123.

KOURI, M., LAASONEN, A., MECKLIN, J.-P., JÄRVINEN, H., FRANSSILA, K. & PYRHÖNEN, S. (1990). Diploid predominance in hereditary nonpolyposis colorectal carcinoma evaluated by flow cytometry. *Cancer*, **65**, 1825.

MECKLIN, J.-P. (1987). Frequency of hereditary colorectal carcinoma. *Gastroenterology*, **93**, 1021.

MELAMED, M.R., ENKER, W.E., BANNER, P., JANOV, A.B., KESSLER, G. & DARZYNKIEWICZ, Z. (1986). Flow cytometry of colorectal of colorectal carcinoma with three-year follow-up. *Dis. Colon Rectum*, **29**, 184.

QUIRKE, P., DIXON, M.F., CLAYDEN, A.D. & 4 others (1987). Prognostic significance of DNA aneuploidy and cell proliferation in rectal adenocarcinomas. *J. Pathol.*, **151**, 285.

ROGNUM, T.O., THORUD, E. & LUND, E. (1987). Survival of large bowel carcinoma patients with different ploidy. *Br. J. Cancer*, **56**, 633.

SCHUTTE, B., REYNDERS, M.M.J., WIGGERS, T. & 4 others (1987). Retrospective analysis of the prognostic significance of DNA content and proliferative activity in large bowel carcinoma. *Cancer Res.*, **47**, 5494.

SCOTT, N.A., RAINWATER, L.M., WIEAND, H.S. & 4 others (1987*a*). The relative prognostic value of flow cytometric DNA analysis and conventional clinicopathologic criteria in patients with operable rectal carcinoma. *Dis. Colon Rectum*, **30**, 513.

SCOTT, N.A., WIEAND, H.S., MOERTEL, C.G., CHA, S.S., BEART, R.W. & LIEBER, M.M. (1987*b*). Colorectal cancer. Dukes' stage, tumor site, preoperative plasma CEA level, and patient prognosis related to tumor DNA ploidy pattern. *Arch. Surg.*, **122**, 1375.

SECKINGER, D., SUGERBAKER, E. & FRANKFURT, O.(1989). DNA content in human cancer. Application in pathology and clinical medicine. *Arch. Pathol. Lab. Med.*, **113**, 619.

SHAPIRO, H.M. (1989). Flow cytometry of DNA content and other indicators of proliferative activity. *Arch. Pathol. Lab. Med.*, **113**, 591.

VINDELØV, L.L., CHRISTENSEN, I.J. & NISSEN, N.I. (1983). Standardization of high-resolution flow cytometric DNA analysis by the simultaneous use of chicken and trout red blood cells as internal standards. *Cytometry*, **3**, 328.

WIGGERS, T., ARENDS, J.W., SCHUTTE, B., VOLOVICS, L. & BOSMAN, F.T. (1988). A multivariate analysis of pathologic prognostic indicators in large bowel cancer. *Cancer*, **61**, 386.

WOLLEY, R.C., SCHREIBER, K., KOSS, L.G., KARAS, M. & SHERMAN, A. (1982). DNA distribution in human colorectal carcinomas and its relationship to clinical behavior. *J. Natl Cancer Inst.*, **69**, 15.